# Hepatectomy for rapidly growing solitary liver metastasis from non-small cell lung cancer: a case report

**DOI:** 10.1186/s40792-019-0633-6

**Published:** 2019-05-02

**Authors:** Hiroyuki Hakoda, Yasuharu Sekine, Hideo Ichimura, Kazumitsu Ueda, Shigeo Aoki, Hideyuki Mishima, Akihiro Sako, Keisuke Kobayashi, Akiko Sakata, Yukio Sato

**Affiliations:** 1 Department of Surgery, Hitachi General Hospital, Hitachi, Japan; 2 Department of Thoracic Surgery, Hitachi General Hospital, Hitachi, Japan; 3 Department of Pathology, Hitachi General Hospital, Hitachi, Japan; 4 Department of Thoracic Surgery, Faculty of Medicine, University of Tsukuba, Tsukuba, Japan; 5 Department of Thoracic Surgery, Faculty of Medicine, University of Tsukuba, Hitachi Medical Education and Research Center, 2-1-1 Jyounan, Hitachi, Ibaraki, 317-0077 Japan

**Keywords:** Metastatic liver tumor, Hepatectomy, Non-small cell lung cancer, Oligo-metastasis, Oligo-recurrence, Tumor doubling time

## Abstract

**Background:**

Patients with liver metastasis from non-small lung cancer (NSCLC) usually have multiple metastases at other sites and thus rarely undergo liver surgery. We present a case involving successful resection of rapidly growing liver metastasis from squamous cell carcinoma of the lung.

**Case presentation:**

A 74-year-old man had undergone left lower lobectomy for squamous cell carcinoma of the lung, which was diagnosed pathologically as stage IA. A computed tomography (CT) scan that was taken 12 months after lung resection showed an irregularly shaped mass lesion (size, 8.3 cm) in segment five of the liver. Retrospectively, the mass was identifiable on CT 6 months before this initial recognition. Although the lesion showed rapid growth, positron emission tomography and brain magnetic resonance imaging ruled out the possibility of other metastatic lesions. Therefore, we performed right hepatectomy 14 months after the initial lung surgery. The patient was pathologically diagnosed with liver metastasis from lung cancer and has remained free from recurrence 41 months after the liver surgery, without receiving any adjuvant chemotherapy.

**Conclusions:**

Although there is no reliable clinical indicator for selecting oligo-recurrence, hepatectomy could be an option for solitary liver metastasis from NSCLC for patients who are in good health.

## Background

Although the recurrences that have been observed after complete resection for non-small cell lung cancer (NSCLC) mostly include multiple organs or sites and are treated with systemic anticancer drugs, oligo-recurrence/metastasis is considered a recurrent mode that could be controlled using a definitive local therapy [1–3]. However, selecting the appropriate patients for definitive local therapy remains an intractable issue.

Since the liver is rarely observed as an oligo-recurrence/metastasis site in patients with NSCLC [1], hepatectomies for liver metastasis from NSCLC have rarely been reported [4]. We encountered a patient with a rapidly growing solitary liver mass lesion that developed after lung resection for NSCLC and treated the patient using a hepatectomy.

## Case presentation

**Fig. 1:**
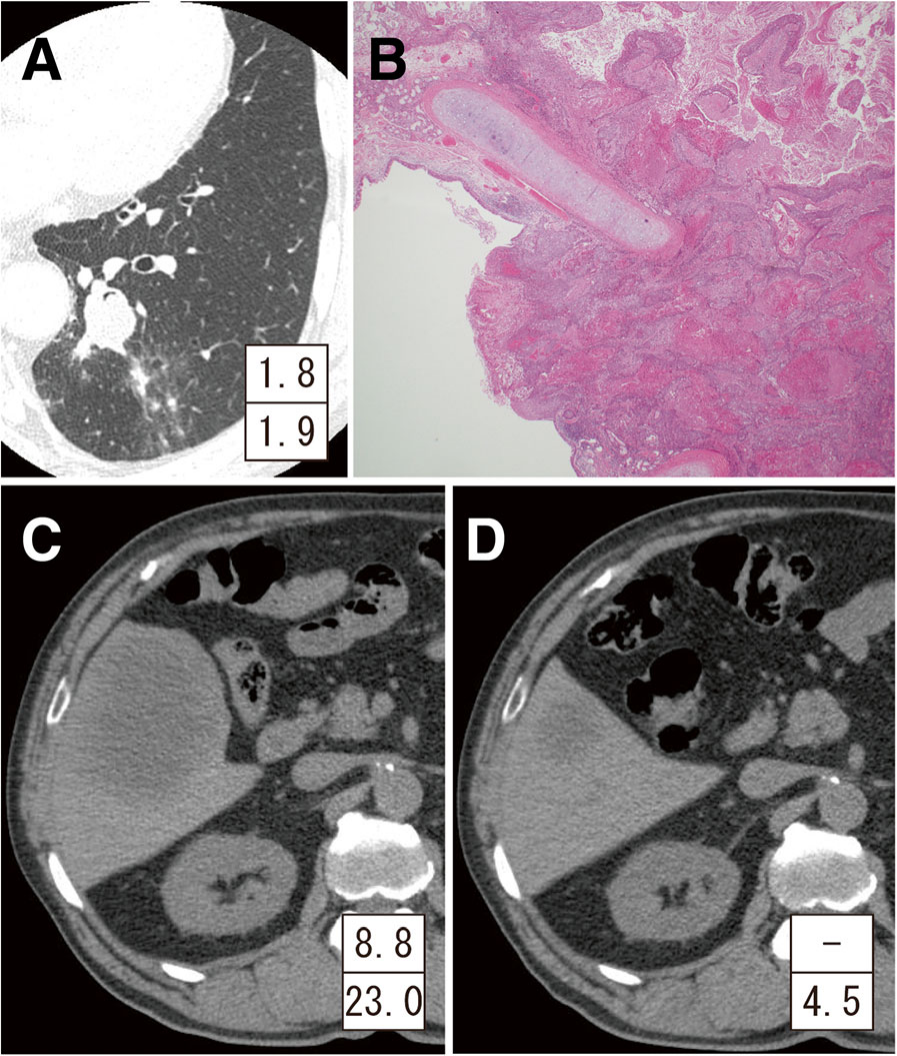
**a** Chest computed tomography (CT) shows 1.8-cm nodule in the left lower lobe of the lung. **b** The pathological image shows squamous cell carcinoma of the lung (hematoxylin-eosin stain). **c** The abdominal CT taken 12 months after lung resection shows an 8.3-cm mass in the right liver. **d** CT taken 6 months after lung resection shows a 3.1-cm low-density lesion in the liver. Insets in **a**, **c**, and **d** show serum levels of tumor markers at the time (upper: carcinoembryonic antigen [CEA]; lower: cytokeratin 19 fragment [CYFRA])

**Fig. 2:**
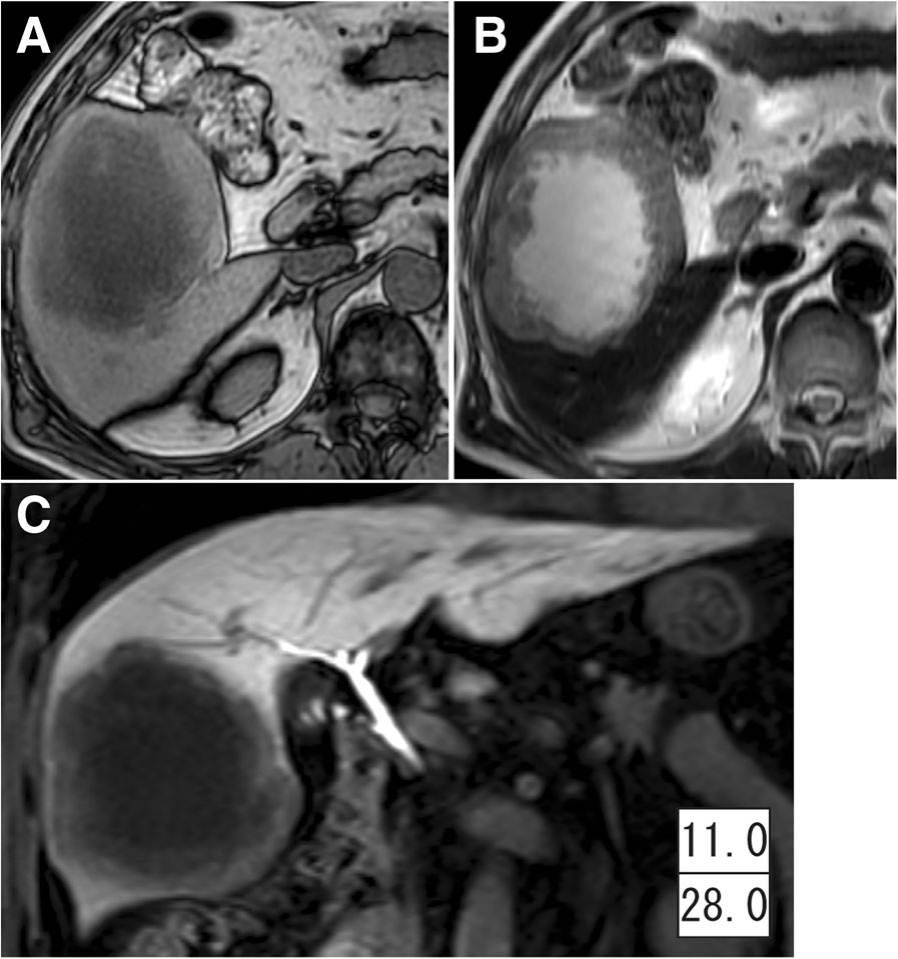
Magnetic resonance imaging demonstrates a large mass with hypointensity on T1-weighted images (**a**) and hyperintensity on T2-weighted images (**b**). The tumor exhibits clear hypointensity in the late dynamic and hepatobiliary phases (**c**). Inset in **c** shows serum levels of tumor markers at the time (upper: carcinoembryonic antigen [CEA]; lower: cytokeratin 19 fragment [CYFRA])

A 74-year-old man had undergone left lower lobectomy for NSCLC (Fig. 1a). The tumor was pathologically diagnosed as squamous cell carcinoma of the lung (1.9 cm in size) without lymph node metastasis (TNM classification 7th edition, pT1aN0M0 and stage IB) (Fig. 1b). He was followed up periodically, and a computed tomography (CT) scan that was taken 1 year after the operation revealed an 8.3 cm, irregularly shaped mass lesion in segment five of the liver (Fig. 1c). Retrospectively, CT performed 6 months prior showed a 3.1 × 2.9 cm low-density lesion at the identical site (Fig. 1d). Since 18-fluoro-2-deoxyglucose (^18^F-FDG) positron emission tomography and enhanced brain magnetic resonance imaging (MRI) ruled out any metastatic lesions other than the one in the liver, the patient was referred to a gastroenterological surgeon. Liver MRI demonstrated a well-defined mass, which was hypointense relative to the liver parenchyma on T1-weighted images (Fig. 2a) and hyperintense on T2-weighted images (Fig. 2b). The hepatic mass exhibited clear hypointensity in the late dynamic and hepatobiliary phases on ethoxybenzyl diethylenetriaminepentaacetic acid-MRI (Fig. 2c). Moreover, MRI showed that the mass had increased to 9.6 cm in diameter within a 1-month interval. Although serum levels of carcinoembryonic antigen (CEA; cut-off value, 3.4 ng/ml) and cytokeratin 19 fragment (CYFRA; cut-off value, 3.5 ng/ml) were both within the normal range at the time of lung resection, both CEA and CYFRA levels increased to 11.0 ng/ml and 23.0 ng/ml, respectively, along with enlargement of the hepatic mass (CEA and CYFRA levels at each time point are indicated in Figs. 1 and 2).

Since his general condition was good and his major organ functions were tolerable to general anesthesia, the patient underwent right hepatectomy 14 months after the lung resection at the primary site. Intraoperatively, a huge mass was detected in the right liver, but no other metastatic sites were identified. The postoperative course was uneventful, and the patient was discharged on postoperative day 10.

**Fig. 3:**
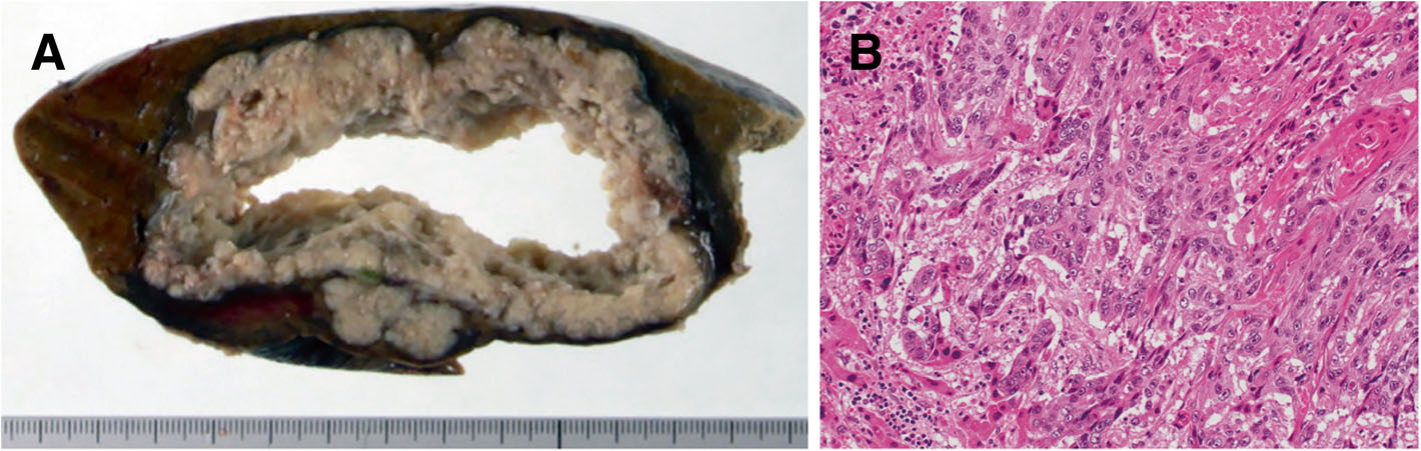
**a** A macroscopic image of the cut specimen shows an irregular, grayish mass measuring 10.0 cm with massive central necrosis. **b** The microscopic image of the liver mass shows squamous cell carcinoma

The macroscopic examination of the cut specimen showed an irregular, grayish mass that measured 10 × 8 × 5.5 cm, with massive central necrosis (Fig. 3a). The pathologic examination confirmed metastatic squamous cell carcinoma of the lung (Fig. 3b). He did not receive adjuvant chemotherapy and was free from recurrence 41 months after the hepatectomy.

## Discussion

**Fig. 4:**
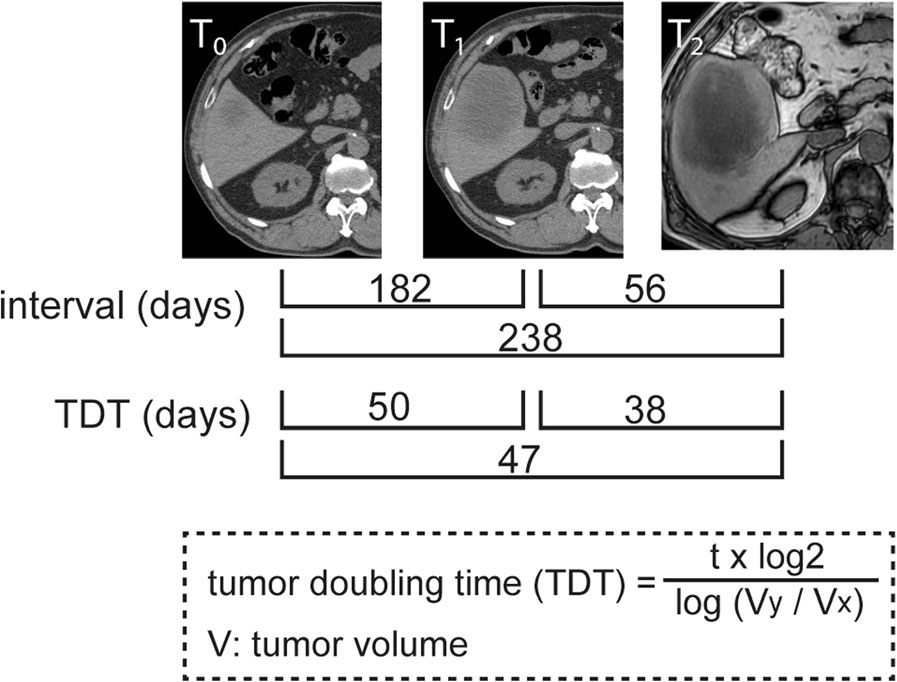
Tumor doubling time (TDT) in the present case between each time point. The indicated formula was described by Hasegawa et al. [5]

Here, we performed a right hepatectomy for a rapidly growing giant liver metastasis from NSCLC, and the patient did not present recurrence 41 months after the hepatectomy and 54 months after the lung resection. Although the liver is not a common recurrence site [3], and reported cases of hepatectomies for liver metastases from NSCLC are rare [4], we recommended surgical resection to this patient. Since the tumor showed substantial growth after we overlooked the liver lesion 6 months before the initial recognition and the new metastatic lesion did not emerge during the 6-month interval, we considered that the liver metastasis could be an oligo-metastasis. Retrospectively, we verified whether our presumption based on an unintentional 6-month observation was supported by the tumor doubling time (TDT) [5]. In this case, the TDT ranged from 38 to 50 days (Fig. 4). If the patient had other clinically unrecognizable metastatic lesions that were 2 mm in diameter and could grow to a recognizable 1 cm diameter, this increase would take 116 days, since the TDT is 50 days. Although 6 months is longer than this 116-day interval, we can assume that a single metastatic cell from this patient would require 30 times the TDT (30 × 50 = 1500 days) bio-mathematically [6] to become a nodule with a diameter of 1 cm. Thus, our presumption that this case truly involves oligo-metastasis is not fully supported by the TDT model. To select appropriate patients with oligo-recurrences/metastases for definitive local therapy, other clinical indicators, such as circulating tumor cells, should be developed [7].

We reviewed hepatectomy cases from the literature available in English and Japanese for reports of metachronous liver metastases from NSCLC, excluding patients who were diagnosed with liver metastasis concurrently with lung cancer, and found 21 cases, including the present one (Table 1) [8–17]. Among these patients, there is a case who is not truly oligo-recurrence because of multiple abdominal lymph node metastases [8]. Moreover, Nagashima et al. [17] mentioned that they did not report two other patients who underwent hepatectomies and did not survive. Although it is impossible to rule out selection and publishing biases in our review of reported cases, the median survival time of all cases after hepatectomy is 24.3 months, and the median interval between initial lung surgery and hepatectomy is 11 months (mean 15 months, range 1.6–48 months). The interval is not significantly different between post-hepatectomy survival and mortality cases (mean 16.8 and 11.4 months, Mann-Whitney *U* test: *p* = 0.40). Squamous cell carcinoma was the dominant histologic type, and this finding is consistent with Hishida et al.’s observation [1], which showed that oligo-recurrence was non-adenocarcinoma histology dominant. On survival analyses, cases of pathological stage I lung cancer showed better post-hepatectomy prognosis than those of pathological stage II or more (2-year survival rate 87.5% and 50.0%, respectively). Moreover, cases with squamous cell carcinoma showed better post-hepatectomy prognosis than those with non-squamous histologies (2-year survival rate 87.5% and 55.6%, respectively). Tumor angiogenesis varies between histologic types [18], and this might affect the recurrent mode and prognosis after definitive local therapy for recurrence.

**Table 1:** Reported cases of hepatectomy for metachronous liver metastases from non-small cell lung cancer

No.	Author	Age	Sex	Primary lesion				Time interval (months)	Liver metastases					
				Management	Stage	Histological type	Adjuvant therapy		Location	Size (cm)	Number	Hepatectomy procedure	Treatment for recurrences	Outcome (months)
1	Di Carlo	69	F	L	NA	AD	–	48	S7	5	1	P	–	Alive (36)
2	Takagi	46	M	L	NA	Pleo	–	10	S6	10	1	S	CT	Dead (12)
3	Nagashima	71	M	L	*IB*	AD	–	6	S5/S6	2.5/0.4	2	P	–	Alive(62)
4	Nikkumi	60	M	L	*IB*	SQ	RT	12	S7	7	1	P	Surg	Alive (73)
5	Kim	55	M	Pn*	*IA*	SQ	–	7	caudate	NA	1	S	–	Alive (60)
6	Ercolani	52	M	L	NA	AD	CT	24	S6	5	1	S	–	Dead(36)
7	Ercolani	60	F	L	NA	AD	CT	18	NA	6	1	L	–	Alive (NA)
8	Ileana	56	M	L*	IIIA	LCNEC	–	24	NA	NA	3	L	CT	Alive(21)
9	Ileana	70	F	CCRT	IIIB	AD	–	9	NA	3	1	NA	CT	Dead (5.5)
10	Ueda	77	M	L	*IIA*	LA	CT, RT	8	S8	5	1	S	–	Alive (6)
11	Ueda	65	F	L	*IIIA*	AD	CT, RT	20	S7	2	1	S	–	Dead (20)
12	Higaki	77	M	P	*IIB*	SQ	–	10	S2	1	1	S	RT, BI, CT	Alive(33)
13	Watanabe	71	M	L	*IA*	AD	–	11	S6	3.5	1	P	EGFRTKI	Alive (6)
14	Ishige	NA	M	Surg	*IIIA*	SQ	–	1.6	NA	4.5	1	S	RT	Dead(15.2)
15	Ishige	NA	M	Surg	*IB*	SQ	–	15	NA	4	4	P	CT	Alive(23.4)
16	Ishige	NA	M	Surg	*IIIA*	SQ	CT	8	NA	4	1	S	CT	Dead(24.5)
17	Ishige	NA	M	Surg	*IA*	SQ	–	7	NA	2.3	3	S	NA	Alive(30.2)
18	Ishige	NA	M	Surg	*IB*	SQ	CT	13	NA	3	1	P	CT	Alive(26.2)
19	Ishige	NA	F	Surg	*IB*	AD	–	42	NA	1.9	4	P	–	Alive(24)
20	Ishige	NA	M	Surg	*IB*	LA	–	7.4	NA	7.5	1	S	–	Dead(16.5)
21	Present case	74	M	L	*IB*	SQ	–	14	S5	9.6	1	L	–	Alive (41)

*NA* not available, *AD* adenocarcinoma, *SQ* squamous cell carcinoma, *LA* large cell carcinoma, *LCNEC* large cell neuroendocrine carcinoma, *Pleo* pleomorphic carcinoma, *CT* chemotherapy, *RT* radiotherapy, *CCRT* concurrent chemoradiotherapy, *time interval* the interval of time between primary lesion treatment and hepatectomy, *P* partial resection, *S* segmentectomy or sectionectomy, *Surg* surgery, *L* lobectomy, *Pn* pneumonectomy, *BI* bronchoscopic intervention, *EGFRTKI* epidermal growth factor receptor tyrosine kinase inhibitor

Italic font indicates the pathological stage

*Induction therapy was administered

## Conclusion

We report on a patient who underwent hepatectomy for liver metastasis from NSCLC and remained recurrence free at 41 months after liver surgery. Although we do not have a reliable clinical indicator for selecting oligo-recurrent cases, hepatectomy provides an option for patients with solitary liver metastasis from NSCLC. Based on our review of reported cases, patients with pathological stage I NSCLC and squamous cell carcinoma who undergo hepatectomies demonstrate better post-hepatectomy prognoses.

## Abbreviations

NSCLC: Non-small cell lung cancer
TDT: Tumor doubling time
